# Inhibitory Control Impairment on Somatosensory Gating Due to Aging: An Event-Related Potential Study

**DOI:** 10.3389/fnhum.2018.00280

**Published:** 2018-07-12

**Authors:** Juan L. Terrasa, Pedro Montoya, Ana M. González-Roldán, Carolina Sitges

**Affiliations:** Cognitive and Affective Neuroscience and Clinical Psychology, Research Institute of Health Sciences (IUNICS), Balearic Islands Health Research Institute, University of the Balearic Islands, Palma, Spain

**Keywords:** aging, somatosensory gating, paired-pulse task, event-related potential, source localization, inhibitory deficit hypothesis

## Abstract

The capacity to suppress irrelevant incoming input, termed sensory gating, is one of the most investigated inhibitory processes associated with cognitive impairments due to aging. The aim of this study was to examine the influence of aging on sensory gating by using somatosensory event-related potentials (ERPs) elicited by repetitive non-painful tactile stimulation (paired-pulsed task). Somatosensory ERPs were recorded in 20 healthy young adults and 20 healthy older adults while they received two identical pneumatic stimuli (S1 and S2) of 100 ms duration with an inter-stimulus interval of 550 ± 50 ms on both forefingers. The difference between the somatosensory ERPs amplitude elicited by S1 and S2 was computed as a sensory gating measure. The amplitude and the latency of P50, N100 and late positive complex (LPC) were analyzed as well as the source generators of the gating effect. Reduced sensory gating was found in older individuals for N100 at frontal and centro-parietal electrodes and for LPC at fronto-central electrodes. Source localization analyses also revealed a reduced current density during gating effect in the older group in frontal areas in N100 and LPC. Moreover, older individuals showed delayed latencies in N100. No significant gating effect differences were found between groups in P50. These findings suggest an age-related slowing of processing speed and a reduced efficiency of inhibitory mechanisms in response to repetitive somatosensory information during stimulus evaluation, and a preservation of processing speed and inhibitory control during early stimulus coding in aging.

## Introduction

Physiological aging is associated with functional impairments affecting psychological processes such as memory, executive functions and attention (Hedden and Gabrieli, 2004; Salthouse, 2011). Although the nature and severity of sensory and cognitive decline in older individuals is heterogeneous (Gazzaley and D'Esposito, 2007), aging could be related to reduced efficiency of inhibitory mechanisms (inhibitory deficit hypothesis) (Hasher and Zacks, 1988). Accordingly, age-related decline in early sensory processing has been associated with changes in sensory cortices, including decreased inhibition at the primary somatosensory cortex (SI) during tactile stimulation of fingertips (Brodoehl et al., 2013) and increased excitability in SI after median nerve stimulation in a paired-pulse task (Lenz et al., 2012). Furthermore, several research have examined the influence of aging on automatic sensory functions (Bertoli et al., 2002; Cheng et al., 2013; Strömmer et al., 2017). Thus, for instance, it has been demonstrated that elderly adults show smaller ERPs amplitudes to an odd stimulus than young adults indicating an impairment in change-detection efficiency. In this regard, sensory gating protocols have been also used to evaluate age-related changes in ERPs amplitudes.

Sensory gating refers to the ability of the central nervous system to inhibit the processing of repetitive and irrelevant sensory inputs which is an essential protective mechanism that avoids the flooding of higher cortical centers (Freedman et al., 1987; Cromwell et al., 2008). Neurophysiological correlates of auditory and somatosensory gating phenomena have been examined by using event-related potentials (ERPs) elicited by repetitive stimuli in paired S1–S2 tasks with short inter-stimulus time intervals (e.g., 500 ms). Sensory gating is computed as the ratio between ERP amplitudes elicited by S2 and S1 stimuli (S2/S1), or as the amplitude difference between the two stimuli responses (S1–S2). Lower ratios or larger differences may indicate better gating or inhibition of irrelevant inputs (Boutros and Belger, 1999). Sensory gating of P50, N100, and P200 amplitudes of the ERPs elicited by auditory stimuli has received considerable interest because of its possible application in clinical research such as schizophrenia (Brenner et al., 2009) and Alzheimer's disease (Jessen et al., 2001). Moreover, it has been shown that chronic pain patients displayed a reduced sensory gating effect in response to repetitive non-painful somatosensory but not to auditory stimuli (Montoya et al., 2006). These ERPs components has been widely studied. Specifically, P50 represents one of the earliest evoked cortical responses to somatic stimulation (Freedman et al., 1987), N100 is assumed to reflect a trigger to allocate attention (Näätänen and Picton, 1987) and the late positive complex (LPC, 150–350 ms) have been associated with memory and stimulus evaluation (Polich and Herbst, 2000). Nevertheless, the evidences of age-related effects on sensory gating of these components and which variables are involved still limited and unclear (Bolton and Staines, 2012; Cheng et al., 2015a). Previous studies have reported significant age-related impairments of auditory gating (Boutros et al., 2000; Kisley et al., 2005), whereas recent studies failed to replicate this effect (Lijffijt et al., 2009; Gmehlin et al., 2011). Further studies using a paired-pulse electrical stimulation of the left median nerve protocol revealed significant higher S2/S1 ratios (Cheng and Lin, 2013) and a disturbed association between SI and the motor cortex (Cheng et al., 2018) in an elderly group compared to a younger group, reflecting an age-related decline of somatosensory gating. Finally, Cheng et al. (2015b) pointed to an altered alpha oscillatory activity in response to somatosensory stimulation as a possible explanation to age-related reduced sensory gating.

The present study was aimed at examining possible age-related impairments in the ability of the brain to inhibit irrelevant non-painful somatosensory information in healthy older adults. For this purpose, event-related potentials elicited by repetitive, paired tactile stimulation of the fingertip, as well as source localization of the sensory gating effect were analyzed. We hypothesized that older subjects would show reduced sensory gating and delayed latencies of the P50, N100, and LPC components compared to younger participants.

## Materials and methods

### Participants

Twenty healthy young adults (18–30 years old, 8 male) and 20 healthy older adults (65–83 years old, 14 male) participated in the study. All participants were right handed. Upon arrival to the lab, participants were interviewed to exclude those subjects with any neurological or psychiatric disorder. All individuals were naive to the experiment, had no history of drug abuse and gave informed consent after the experimental procedure was explained. Twenty-five percent of the older adults were diagnosed and medicated as hypertensives. The study was conducted in accordance with the Declaration of Helsinki (1991) and was approved by the Ethics Committee of the University of Balearic Islands. Subjects completed the Spanish versions of the Beck Depression Inventory (BDI-II) (Beck et al., 1996), the State-Trait Anxiety Inventory (STAI) (Spielberger et al., 1970) and the Edinburgh Handedness Inventory (EHI) (Oldfield, 1971).

### Measurement of blood pressure and pressure pain thresholds

In order to control confounding variables and based on previous research (Cicconetti et al., 2007; Martínez-Jauand et al., 2013), pressure pain thresholds and blood pressure were measured before starting the main experiment. Blood pressure was measured twice in the right arm with a tensiometer (OMRON MX2, OMRON Healthcare, Hoofddorp, Netherlands) after the participant was seated and after they had rested for 5 min. Pressure pain thresholds were also assessed twice with a digital dynamometer using a flat rubber tip (1cm^2^, Force One, Wagner Instruments, Greenwich, CT USA) at three body locations: index fingertip, medial area of the ventral surface of the wrist and dorsal area of the shoulder, measured always in this order. All locations were chosen as pain-free sites. The pressure pain threshold was defined as the amount of pressure in Newtons (N) at which participants perceived the pressure stimulus as painful. Participants were asked to rate the subjective pain sensation of the stimulus in a 100-point numerical scale (0: no pain, 100: maximum pain). For safety reasons, the maximal force allowed was 140 N. The mean of the two measurements on each body location was used in the statistical analysis.

### Non-painful paired-pulse stimulation task

In the main experiment, subjects received tactile paired-pulse stimulation to analyze the sensory gating mechanism. For this purpose, stimulation consisted of two identical non-painful pneumatic stimuli (S1 and S2) of 100 ms duration that were delivered to both forefingers. Stimulation was applied with a constant pressure of 2 bars, a randomized inter stimulus interval of 550 ms (± 50 ms) and separated by a fixed interval of 12 s. The pneumatic stimulator was used in previous research (e.g., Montoya et al., 2006) and consisted of a small membrane attached to the body surface by a plastic clip and fixated with adhesive strips. Forty trials were presented in a single run session by using Presentation software (Version 18.3, Neurobehavioral Systems, Inc., Berkeley, CA, USA, www.neurobs.com).

### EEG recording and data reduction

EEG was recorded with a commercial amplifier (QuickAmp, Brain Products GmbH, Munich, Germany) at 1,000 Hz sampling rate from 46 Ag/AgCl electrodes placed according of the 10–10 International System. A ground electrode was located at position AFz. A common average reference was used. An electroculogram (EOG) was also recorded by placing one electrode above and one below the left eye. Electrode impedances were kept lower than 10 kOhm.

During data pre-processing, EEG signals were segmented in epochs of 500 ms (−100 to 400 ms relative to the stimulus onset) and were digitally filtered (high-pass at 0.10 Hz, low-pass at 30 Hz, notch filter at 50 Hz) and baseline corrected (from −100 to 0 ms). Eye movement artifacts were corrected by using Gratton & Coles algorithm (Gratton et al., 1983). Then, an artifact rejection protocol with the following criteria was applied: maximal allowed voltage step/sampling point = 100 mV, minimal allowed amplitude = −100 mV, maximal allowed amplitude = 100 mV, and maximal allowed absolute difference in the epoch = 100 mV. EEG epochs were separately averaged for S1 and S2. In addition, the difference between the amplitude evoked by the first in comparison to the second stimuli (S1–S2) was computed, as gating score measure. Finally, somatosensory ERP amplitudes were determined by using a global maxima detection method and searching separately for each channel in three different time windows after stimulus onset: 20–80 ms (P50), 70–150 ms (N100) and mean amplitude during 150–350 ms (late positive complex, LPC). Peak latencies of P50 and N100 components were calculated for each electrode location.

### Data analyses

Group differences on gender were analyzed with Chi-Squared Test. The rest of sociodemographic data, self-reports, blood pressure and pressure pain thresholds were analyzed with Student *t*-tests. Regarding EEG, data from 12 electrodes were grouped and statistically analyzed into four regions of interest: frontal (F3, Fz, and F4), fontro-central (FC3, FCz, and FC4), central (C3, Cz, and C4) and centro-parietal (CP3, CPz, and CP4). Two separate multivariate analyses of variance (MANOVA) with repeated-measures were performed using “group” (young vs. older) as between-subject factor and “location” (4 regions) as within-subject factor on P50, N100, and LPC amplitudes. In the first analysis, the “stimulus type” (S1 vs. S2) was used as an additional within-subject factor. In the second analysis, MANOVAs were performed on the amplitude difference elicited by S1 minus S2. Greenhouse-Geisser adjustments were applied and *post-hoc* Bonferroni corrected paired tests were used when necessary. Finally, Spearman's correlations between age and S1, S2 and S1–S2 amplitude difference for each ERP component were also computed.

### Source localization

Source localization was computed by using sLORETA (Pascual-Marqui, 2002). This software gives a single linear solution to the inverse problem of localization of brain function and produces images of standardized current density with no localization bias. The localization accuracy of sLORETA has been validated in EEG/fMRI studies (Vitacco et al., 2002; Mulert et al., 2004). For sLORETA, the intracerebral volume is partitioned in 6,239 voxels at 5 mm spatial resolution, and the standardized current density at each voxel is then calculated in a realistic head model (Fuchs et al., 2002) using the MNI152 template (Mazziotta et al., 2001). In the present study, the voxel-based data were created from the difference waveforms elicited by S1 and S2 stimuli as a sensory gating measure. Then, the sLORETA images for P50, N100, and LPC were generated by comparing the sensory gating scores (S1–S2) in the young group with respect to the older group (paired-sample *t*-tests). Voxels with significant group differences (*p* < 0.01) were located in the MNI-brain and Broadmann areas.

## Results

### Clinical and sociodemographic data

Table 1 displays clinical and sociodemographic data of both groups. Student *t*-test analyses revealed significant differences on age [*t*_(38)_ = −31.824, *p* < 0.001] between young (23.14 ± 3.24 years) and older (69.30 ± 5.56 years) groups as expected, but also on gender [$\chi_{\left(1,40\right)}^{2}$ = 4.912, *p* < 0.05], systolic blood pressure [*t*_(38)_ = −5.728, *p* < 0.001] and diastolic blood pressure [*t*_(38)_ = −3.721, *p* < 0.01]. Both blood pressure measures were higher in the older group. No significant group differences were found on BDI-II, STAI, pressure pain thresholds or subjective pain ratings.

**Table 1:** Clinical and sociodemographic data of the participants in each group.

		**Young (*n* = 20) Mean (SD)**	**Older (*n* = 20) Mean (SD)**	***t*-value**	***p***
Age (years)		23.2 (3.318)	69.3 (5.564)	−31.824	*p* < 0.001
Gender (males)		8	14	4.912^†^	*p* < 0.05
Blood pressure (mm Hg)	Systolic	11 (1.379)	13.9 (1.771)	−5.718	*p* < 0.001
	Diastolic	7.2 (1.189)	8.5 (0.967)	−3.721	*p* < 0.01
BDI (0–63)		6.6 (4.893)	10.6 (7.639)	−1.972	*p* = 0.056
STAI (0–60)	State	13.7 (7.419)	11.8 (8.475)	0.754	*p* = 0.455
	Trait	20.1 (9.349)	15.3 (8.738)	1.673	*p* = 0.102
EHI (10–50)		16.7 (3.011)	16.4 (3.605)	0.238	*p* = 0.813
Pain threshold (N)	Finger	107.8 (23.971)	111.4 (25.604)	−0.458	*p* = 0.649
	Wrist	94.6 (27.041)	102.1 (24.880)	0.913	*p* = 0.938
	Shoulder	91.4 (30.744)	105.3 (27.039)	−1.516	*p* = 0.367
Pain rating (0–100)	Finger	29.5 (24.704)	30.2 (29.675)	−0.078	*p* = 0.762
	Wrist	38.6 (25.255)	41.2 (29.104)	−0.305	*p* = 0.138
	Shoulder	42.4 (22.905)	35.7 (29.693)	0.790	*p* = 0.434

*Means and standard deviations (SD) are shown. BDI, Beck Depression Inventory; STAI, State-Trait Anxiety Inventory; EHI, Edinburgh Handedness Inventory*.

† *Chi-Squared value*.

### Somatosensory ERP amplitudes

**Figure 1:**
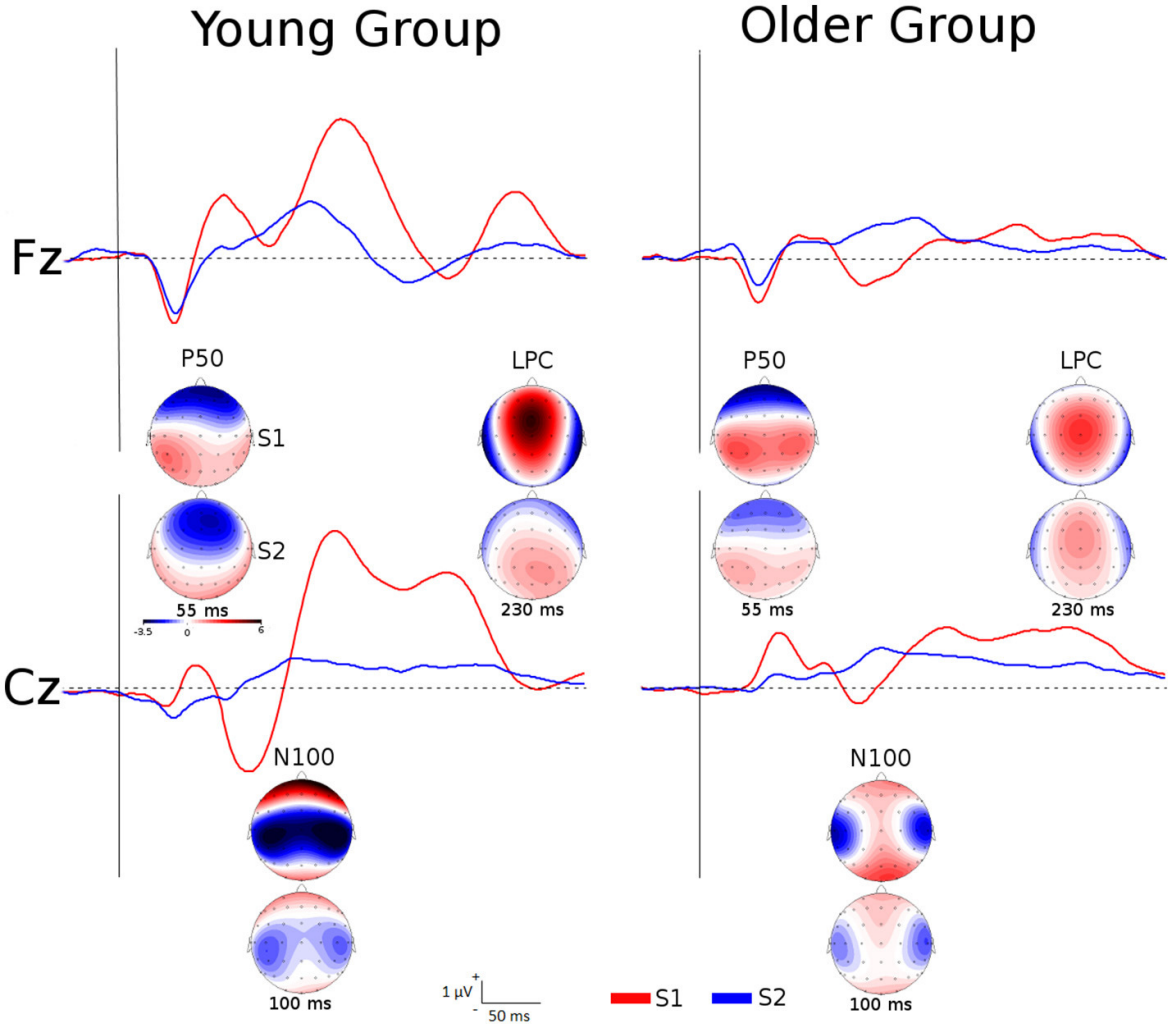
Grand averages of the somatosensory ERPs elicited by S1 and S2 at Fz and Cz for each group as well as topographic maps of P50, N100, and LPC amplitudes at specific latencies.

Table 2 displays the grand averages of the somatosensory ERPs elicited by the first (S1) and the second stimuli (S2) at the four regions of interest for each group. Figure 1 illustrates these ERPs at Fz and Cz. For P50, a main effect of *group* [*F*_(1, 35)_ = 11.932, *p* < 0.01, ηp^2^ = 0.254] revealed a greater response amplitude in older (1.852 ±.596 μV) than in young individuals (1.620 ±.548 μV). Furthermore, a significant interaction effect of *location x stimuli* [*F*_(3, 114)_ = 112.290, *p* < 0.001, ηp^2^ = 0.747] were found. *Post-hoc* analyses revealed that P50 amplitudes to S2 were significantly reduced compared to S1 at the four regions of interest (frontal, fronto-central, central and centro-parietal) (all *p* < 0.001) in all participants. For N100, significant interaction effects of *location* × *group* [*F*_(3, 114)_ = 12.534, *p* < 0.001, ηp^2^ = 0.248] and *location* × *stimuli* × *group* [*F*_(3, 114)_ = 12.118, *p* < 0.001, ηp^2^ = 0.242] were found. *Post-hoc* analyses showed that N100 amplitudes to S2 were significantly reduced compared to S1 stimuli in young individuals at the four regions of interest (all *p* < 0.001), as well as in the older group at fronto-central and central regions (all *p* < 0.001). Furthermore, N100 amplitudes in response to S1 were significantly reduced in the older when compared to the young group at frontal, central and centro-parietal regions (all *p* < 0.05); whereas N100 amplitudes in response to S2 were significantly reduced only at the fronto-central region (*p* < 0.05). Finally, significant differences due to *group* [*F*_(1, 38)_ = 9.770, *p* < 0.01, ηp^2^ = 0.205] and to *stimuli* × *group* [*F*_(1, 114)_ = 12.220, *p* < 0.01, ηp^2^ = 0.243] were found on LPC amplitudes. *Post-hoc* analyses showed that LPC amplitudes in response to S2 were significantly reduced compared to S1 stimuli only in the young individuals (*p* < 0.001). In addition, LPC amplitudes in response to S1 stimuli were significantly reduced in the older when compared to the young group (*p* < 0.01).

**Table 2:** Mean amplitude in μV and standard deviation (SD) of the three ERP components in response to the first (S1) and second (S2) stimuli in each region of interest and group.

	**Region**	**Young (*****n*** = **20) Mean (SD)**		**Older (*****n*** = **20) Mean (SD)**	
		**S1**	**S2**	**S1**	**S2**
P50	Frontal	−3.18 (1.481)	−1.09 (0.825)	−2.72 (0.964)	−0.68 (0.748)
	Fronto-central	1.45 (1.103)	0.49 (0.456)	1.63 (0.701)	0.94 (0.568)
	Central	1.92 (0.850)	0.59 (0.719)	2.75 (1.105)	1.05 (0.581)
	Centro-parietal	2.07 (1.019)	1.01 (0.813)	2.76 (1.022)	1.28 (0.751)
N100	Frontal	5.45 (3.233)	2.40 (1.159)	2.20 (1.585)	1.73 (0.974)
	Fronto-central	−3.52 (2.236)	−1.51 (0.602)	−3.16 (1.939)	−1.01 (0.655)
	Central	−5.18 (2.523)	−2.04 (0.994)	−3.39 (2.814)	−1.48 (0.951)
	Centro-parietal	−4.14 (2.480)	−1.79 (1.037)	−1.54 (2.126)	−1.23 (0.958)
LPC	Frontal	1.61 (1.587)	0.26 (0.803)	0.30 (1.491)	0.51 (0.772)
	Fronto-central	2.55 (1.595)	0.64 (0.745)	0.86 (1.480)	0.61 (0.718)
	Central	2.24 (1.691)	0.66 (0.657)	0.90 (1.270)	0.41 (0.560)
	Centro-parietal	1.46 (1.575)	0.49(0.678)	1.01 (1.182)	0.23 (0.706)

**Figure 2:**
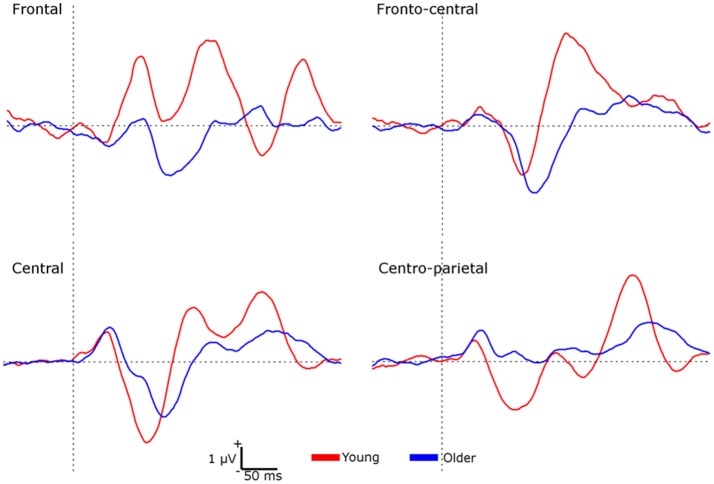
Waveforms representing the sensory gating (S1 minus S2) at each region of interest for each group.

A second group of analyses was performed by using the amplitude difference elicited by S1 and S2 (S1 minus S2) as a sensory gating measure (Figure 2). No significant effects were found on P50 amplitudes. For N100 amplitudes, a significant interaction effect of *location* × *group* [*F*_(3, 114)_ = 12.118, *p* < 0.001, ηp^2^ = 0.242] was found, indicating that sensory gating was significantly higher in the young than in the older group at frontal and centro-parietal locations (all *p* < 0.01). For LPC amplitudes, a main effect of *group* [*F*_(1, 38)_ = 12.220, *p* < 0.01, ηp^2^ = 0.243] revealed a greater gating effect in young (1.452 ± 1.684 μV) than in older individuals (0.323 ± 1.246 μV). Given that the two groups differed on gender, systolic and diastolic blood pressure, multivariate analyses of covariance (MANCOVAs) with repeated-measures were also performed on ERP amplitudes elicited by the S1-S2 difference (sensory gating measure). Similar results were obtained on N100 and LPC amplitudes after controlling for gender and blood pressure.

Finally, correlational analyses revealed that age was negatively correlated with N100 amplitudes elicited by S1 (*r* = −0.483, *p* < 0.01), S2 (*r* = −0.414, *p* < 0.01) and S1–S2 (*r* = −0.438, *p* < 0.01), as well as with LPC amplitudes elicited by S1 (*r* = −0.453, *p* < 0.01) and S1–S2 (*r* = −0.370, *p* < 0.05). No significant correlations between age and P50 amplitudes were found.

### Somatosensory ERP latencies

Regarding peak latencies (Table 3), a main effect of *group* [*F*_(1, 38)_ = 16.948, *p* < 0.001] was found on N100 showing delayed latencies in the older (126.22 ± 8.76 ms) compared to the young group (112.64 ± 5.88 ms). No significant group differences were observed on P50 latencies. Similar results were obtained after controlling for gender, systolic and diastolic blood pressure on P50 and N100 latencies.

**Table 3:** Mean latencies in ms and standard deviation (SD) of the gating effect of P50 and N100 component in each region of interest and group.

	**Region**	**Young (*n* = 20) Mean (SD)**	**Older (*n* = 20) Mean (SD)**
P50	Frontal	61.60 (19.682)	53.90 (16.339)
	Fronto-central	55.18 (13.792)	57.15 (7.946)
	Central	50.53 (7.994)	60.38 (6.479)
	Centro-parietal	51.98 (10.369)	62.82 (8.225)
N100	Frontal	107.85 (25.612)	122.72 (25.947)
	Fronto-central	117.73 (14.259)	134.92 (15.008)
	Central	112.53 (11.063)	131.90 (13.603)
	Centro-parietal	112.43 (17.933)	115.33 (21.798)

### Source localization data

**Figure 3:**
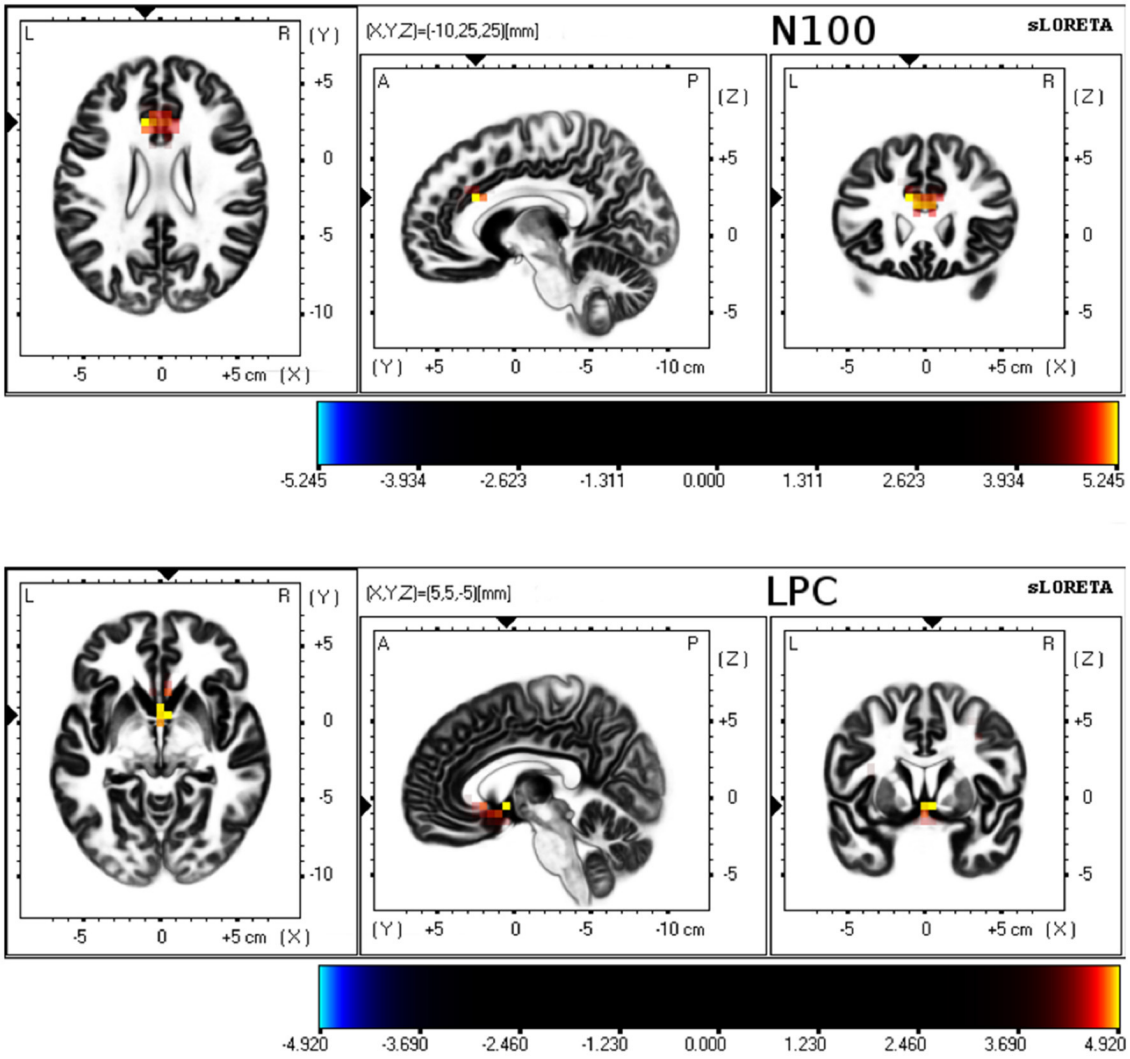
sLORETA results for 3 orthogonal brain slices (horizontal, sagittal, coronal) of N100 and LPC. Colored voxels represent increased (*p* < 0.01) current density of the sensory gating (S1–S2 difference) in young group compared to older group.

Differences between young and older groups on statistical maps of source analyses of each somatosensory ERP component are displayed in Table 4 and Figure 3. Those analyses revealed a significant higher current density during sensory gating in the young group in frontal areas in N100 and LPC components. In particular, N100 component showed higher current density within anterior cingulate cortex (BA 32, BA 33, BA 24), cingulate gyrus (BA 32, BA 24) and medial frontal gyrus (BA 9). LPC component exhibited higher current density within anterior cingulate cortex (BA 25, BA 32, BA 24), precentral gyrus (BA 4, BA 9), insula (BA 9), medial frontal gyrus (BA 6), subcallosal gyrus (BA 25) and postcentral gyrus (BA 40, BA 3, BA 2). No significant group differences were observed on the P50 component.

**Table 4:** Summary of significant results from whole-brain sLORETA comparisons between young and older groups for N100 and LPC components.

**Lobe**	**Region**	**BA**	***X***	***Y***	***Z***
**N100**					
Limbic	Anterior cingulate	32	−10	25	25
	Anterior cingulate	33	−5	20	20
	Anterior cingulate	24	−5	25	20
	Cingulate gyrus	32	−10	30	30
	Cingulate gyrus	24	−10	15	30
Frontal	Medial frontal gyrus	9	10	35	30
**LPC**					
Limbic	Anterior cingulate	25	5	5	−5
	Anterior cingulate	32	5	20	−10
	Anterior cingulate	24	5	25	−5
Frontal	Precentral gyrus	4	−40	−20	40
	Precentral gyrus	9	35	5	40
	Medial frontal gyrus	6	40	0	50
	Subcallosal gyrus	25	0	10	−15
	Postcentral gyrus	40	−40	−30	50
	Postcentral gyrus	3	−40	−25	40
	Postcentral gyrus	2	−35	−30	45
Sublobar	Insula	13	−35	−5	20

*Significant (p < 0.01) regions are indicated with the name of Brodmann area (BA) and MNI coordinates of the higher statistical two-tailed threshold (T) voxel*.

## Discussion

The present study was aimed to testing the effects of aging on sensory gating elicited by repetitive tactile stimulation by using a paired-pulse paradigm and the recording of somatosensory event-related potentials (ERPs). As expected, we found that ERP amplitudes were significantly attenuated when the same stimulus was repeated within a short time interval. Although this gating effect was found in both young and older participants, its magnitude was significantly reduced in older compared to young groups, as shown by N100 and LPC results. Furthermore, it is important to take into account that reduced N100 and LPC amplitudes in response to S1 at some regions in the older group could play a role in the gating impairment. These altered amplitudes may be due to an age-related reduced sensory response (Bourisly, 2016) and/or a higher detection threshold for non-painful somatosensory stimuli (electrical and vibration) due to aging (Lin et al., 2005; Leong et al., 2010). In this sense, Strömmer et al. (2017) reported higher P50 and N80 amplitudes in older compared to young adults in response to somatosensory stimulus. However, these differences disappeared after controlling for the individual stimulus intensities, pointing out that the group differences were due to higher stimulus intensities in older than in young adults rather than related to aging. In contrast, we delivered pressure stimuli with the same intensity for all the participants and we reported that the stimuli (S1 and S2 jointly) elicited higher amplitude response in older than in young participants in P50. This might indicate that the age-related effect found in N100 and LPC is indeed related to sensory gating.

Regarding the gating analyses, no age-related effects on sensory gating were found in P50 component (even after controlling by gender and blood pressure). Source localization of P50 component further revealed no group differences. The P50 component represents one of the earliest evoked cortical responses to somatic stimulation (Freedman et al., 1987), and P50 gating is considered an inhibitory filter mechanism that could protect the integrity of higher-order functions (Wan, 2008). Our results are consistent with previous studies about P50 component in auditory paired -click ERPs. For example, (Gmehlin et al., 2011) reported that the inhibition of recurrent acoustic stimuli in this component did not differ between young and older groups. Moreover, another study revealed that no age-related effects on P50 sensory gating in healthy subjects were found (Lijffijt et al., 2009). These findings suggest that physiological aging is not related with a loss of sensory gating capability in preattentive stages of the sensory responses.

By contrast, N100 gating at frontal and centro-parietal locations were significantly impaired in older participants compared to young participants. We also found that response to both S1 and S2 elicited significantly reduced N100 amplitudes in older as compared to young participants over several brain regions. In addition, N100 amplitudes to both single stimuli and to the S1-S2 difference were negatively correlated with age. These results are also in accordance with previous studies using auditory stimulation (Cooper et al., 2006). In auditory protocols, the N100 response is elicited by perceived sensory stimuli regardless of whether they were attended or not (Näätänen and Picton, 1987; Eimer and Forster, 2003). Our results with a passive stimulation task revealed comparable outcome than previous studies using oddball attentional protocols. Thus, it seems that the relation between age-related changes and N100 gating amplitude could be independent of the task complexity and of the attention required. N100 is assumed to reflect a physiological marker of attention and seems to be generated within the secondary somatosensory cortex and distributed mostly over fronto-central regions (Näätänen and Picton, 1987). Our source localization analyses revealed lower current densities during the gating effect in frontal lobe areas as the medial frontal gyrus and in the anterior cingulate cortex in the older than in the young group. Similar results were reported when auditory S1 and S2 amplitude difference were used (Zhang et al., 2011). These regions have been associated with attention and self-related processing (Posner and Rothbart, 1998; Qin and Northoff, 2011). Moreover, the anterior cingulate cortex participates in a salience network that facilitates the detection of relevant stimuli (Seeley et al., 2007). Therefore, the reduced activity over these brain areas could play a role in the age-related impairment at the attentional component of sensory gating regardless the used sensory modality.

Similar to our N100 findings, older participants displayed an impaired gating effect on LPC amplitudes. Furthermore, LPC amplitudes in response to S1 as well as to the difference S1-S2 (sensory gating) were negatively correlated with age. This sustained late positive amplitude during the time range around 150–350 ms have been associated with more complex cognitive functioning, such as memory or stimulus evaluation (Polich and Herbst, 2000). Our source localization analyses further revealed reduced current densities during the gating effect in older participants over several brain regions areas (precentral and postcentral gyri, anterior cingulate cortex and insula). It has been previously shown that these regions could be relevant brain generators of LPC (P200, P300) during auditory stimulation (Rusiniak et al., 2013; Annic et al., 2016). Again, these findings are in accordance with previous studies with auditory paired-click stimuli demonstrating age-related P200 gating deficits (Boutros et al., 2000; Lijffijt et al., 2009). Our results further suggest that these areas were also associated with age-related gating effects during processing of somatosensory information.

Taking together, our findings about an altered sensory gating effect in healthy older adults compared with younger adults fits with the inhibitory deficit hypothesis, which suggests a reduction in the efficiency of inhibitory mechanisms due to physiological aging (Hasher and Zacks, 1988). These gating impairments were observed during the attentional evaluation of the somatosensory processing and did not appear to be related with early coding of somatosensory information.

Regarding ERP latencies, we found that older participants had longer N100 peak latencies elicited by the S1–S2 difference waves than younger participants at all electrode locations. This finding is in agreement with previous research, showing significant age-related prolongation of N100 latencies and other mid-latency ERP components in auditory (Iragui et al., 1993; Anderer et al., 1996), visual (Dustman et al., 1993; Daffner et al., 2005) and somatosensory processing (Bolton and Staines, 2012). By contrast, we found no significant age-related delays on P50 sensory gating at any electrode locations. Thus, our findings may suggest a significant slowing of attention-related processes due to aging, together with intact early somatosensory processing of repetitive tactile stimulation. This outcome also support the processing speed hypothesis, in which aging-related performance deficits could be attributed to a generalized slowing of processing speed (Salthouse, 1996).

Some limitations of our study merit further consideration. First, 25% of our older participants were diagnosed and medicated as hypertensives. To avoid the possible effects of these drugs on somatosensory ERP response or on the sensory gating process, we repeated the analyses without the hypertensives participants of the older group (see Supplementary Material). The significant effects obtained in the analyses with all the participants were maintained. Thus, hypertension and its medication appear to be irrelevant in the response to stimuli and in the gating effect group differences. Furthermore, although gender and blood pressure differed between groups, the age-related effects of sensory gating on N100 and LPC components were obtained after controlling for those variables. Future research should further explore the role of these variables on brain correlates of somatosensory gating.

In summary, we found a reduced sensory gating to tactile stimuli on mid and late (but not early) stages of information processing in older participants. These findings might indicate: (1) an age-related slowing of processing speed and a reduced efficiency of inhibitory mechanisms in response to repetitive somatosensory information during cognitive evaluation, and (2) a preservation of processing speed and inhibitory control during early stimulus coding in aging.

## Author contributions

JT, PM, AG-R, and CS contributed significantly to the design of the study and analysis. JT did the data collection and wrote most of the manuscript but PM, AG-R, and CS critically revised important parts of the manuscript.

### Conflict of interest statement

The authors declare that the research was conducted in the absence of any commercial or financial relationships that could be construed as a potential conflict of interest.
