# Corrigendum: Inhibitory Control Impairment on Somatosensory Gating Due to Aging: An Event-Related Potential Study

**DOI:** 10.3389/fnhum.2018.00333

**Published:** 2018-08-21

**Authors:** Juan L. Terrasa, Pedro Montoya, Ana M. González-Roldán, Carolina Sitges

**Affiliations:** Cognitive and Affective Neuroscience and Clinical Psychology, Research Institute of Health Sciences (IUNICS), Balearic Islands Health Research Institute, University of the Balearic Islands, Palma, Spain

**Keywords:** aging, somatosensory gating, paired-pulse task, event-related potential, source localization, inhibitory deficit hypothesis

There is an error in the Funding statement. The correct number for PSI2016-78637-P is PSI2016-78637-P AEI/FEDER, UE. In addition, the correct number for PSI2015-66295-R is PSI2015-66295-R AEI/FEDER, UE. The authors apologize for this error and state that this does not change the scientific conclusions of the article in any way.

The original article has been updated.

## Conflict of interest statement

The authors declare that the research was conducted in the absence of any commercial or financial relationships that could be construed as a potential conflict of interest.

