# Supplementary material for: Inhibitory Control Impairment on Somatosensory Gating Due to Aging: An Event-Related Potential Study
Source: Front Hum Neurosci. 2018 Jul 12;12:280. doi: 10.3389/fnhum.2018.00280 (PMC6052091; doi:10.3389/fnhum.2018.00280)
Supplement: Supplementary file 1 [file Data_Sheet_1.DOCX]

Supplementary Material

INHIBITORY CONTROL IMPAIRMENT ON SOMATOSENSORY GATING DUE TO AGING: AN EVENT-RELATED POTENTIAL STUDY

**Juan L Terrasa^1^, Pedro Montoya^1^, Ana M González-Roldán^1^*, Carolina Sitges^1^**

1: Cognitive and Affective Neuroscience and Clinical Psychology, Research Institute of Health Sciences (IUNICS) and Balearic Islands Health Research Institute (IdISBa), University of the Balearic Islands (UIB), Palma, Spain.

***Correspondence:**Ana María González-Roldán, PhD
anamaria.gonzalez@uib.es

# Supplementary Data

To avoid the possible effects of hypertensive medication on somatosensory ERP response or on the sensory gating process, we repeated the analyses without the five hypertensives participants of the older group. Two separate MANOVAs with repeated-measures using “group” (young vs. older) as between-subject factor and “location” (4 regions) as within-subject factor on P50, N100 and LPC amplitudes. In the first analysis, the “stimulus type” (S1 vs. S2) was used as an additional within-subject factor. In the second analysis, MANOVAs were performed on the amplitude difference elicited by S1 minus S2.

First analysis: *Supplementary Table 1* displays the grand averages of the somatosensory ERPs elicited by the first (S1) and the second stimuli (S2) at the four regions of interest for each group. For P50, a main effect of group (F_1,33_=11.536, p<.01, ηp^2^=.262) was found. For N100, significant interaction effects of location x group (F_3,99_=14,380, p<.001, ηp^2^=.304) and location x stimuli x group (F_3,99_=10.592, p<.001, ηp^2^=.243) were found. Finally, significant differences due to group (F_1,33_=12.718, p<.01, ηp^2^=.278) and to stimuli x group (F_1,99_=11.111, p<.01, ηp^2^=.252) were found on LPC amplitudes.

Second analysis: P50 amplitude showed no significant differences. For N100 amplitudes, a significant interaction effect of location x group (F_3,99_=10.592, p<.001, ηp^2^=.243) was found. For LPC amplitudes, a main effect of group (F_1,33_=11.111, p<.01, ηp^2^=.252) was found.

# Supplementary Figures and Tables

**Supplementary Table 1.** Mean amplitude in µV and standard deviation (SD) of the three ERP components in response to the first (S1) and second (S2) stimuli in each region of interest and group.

|  | **Region** | **Young (n=20) Mean(SD)** | | **Older (n=15) Mean(SD)** | | |
| --- | --- | --- | --- | --- | --- | --- |
|  |  | **S1** | **S2** | **S1** | **S2** | |
| **P50** | **Frontal** | -3.18 (1.481) | -1.09 (.825) | -2.75 (1.102) | -1.66 (1.080) | |
|  | **Fronto-central** | 1.45 (1.103) | .49 (.456) | 1.41 (.606) | .91 (.467) | |
|  | **Central** | 1.92 (.850) | .59 (.719) | 2.48 (.921) | .98 (.405) | |
|  | **Centro-parietal** | 2.07 (1.019) | 1.01 (.813) | 2.64 (1.072) | 1.28 (.781) | |
| **N100** | **Frontal** | 5.45 (3.233) | 2.40 (1.159) | 2.00 (1.632) | 1.60 (.818) | |
|  | **Fronto-central** | -3.52 (2.236) | -1.51 (.602) | -2.91 (1.871) | -.89 (.718) |  |
|  | **Central** | -5.18 (2.523) | -2.04 (.994) | -3.09 (2.177) | -1.34 (.780) |  |
|  | **Centro-parietal** | -4.14 (2.480) | -1.79 (1.037) | -1.24 (1.633) | -1.04 (.656) |  |
| **LPC** | **Frontal** | 1.61 (1.587) | .26 (.803) | .48 (1.501) | .47 (.554) |  |
|  | **Fronto-central** | 2.55 (1.595) | .64 (.745) | .81 (1.513) | .52 (.585) |  |
|  | **Central** | 2.24 (1.691) | .66 (.657) | .65 (1.130) | .31 (.464) |  |
|  | **Centro-parietal** | 1.46 (1.575) | .49(.678) | .62 (.564) | .20 (.724) |  |
